# Double-diffusive convection in Jeffery–Hamel flow

**DOI:** 10.1038/s41598-022-12908-9

**Published:** 2022-06-01

**Authors:** Noureen, Dil Nawaz Khan Marwat

**Affiliations:** Department of Mathematics, Faculty of Technologies and Engineering Sciences, Islamia College Peshawar, University Campus, Jamrod Road, Peshawar, Khyber Pakhtunkhwa 25120 Pakistan

**Keywords:** Mathematics and computing, Applied mathematics

## Abstract

In this paper, double-diffusive convection in flow of viscous fluid is investigated inside a horizontal channel. It has heated, inclined and rectangular plane walls. The upper wall has non-uniform temperature and variable species concentration. Note that the Jeffery–Hamel flow depends upon the radial component of velocity, whereas, the peripheral velocity is taken zero. However, the current simulation has been accomplished in view of new procedures and we dealt with two non-zero components of velocity. The problem has been described in a set of four PDEs and the relevant BCs, whereas, the whole set of BVP is taken in Cartesian Coordinates. A set of proper transformation is formed, which reduces the system of PDEs into a new system of ODEs. The system of ODEs is solved with the help of several methods in order to check the validity of the solution. An approximate analytical solution is provided for small values of inclination parameter. An accurate numerical solution of the modelled equations is also given. Moreover, skin friction, rate of the two diffusions are investigated for all different cases of assisting (opposing) and converging (diverging) flows. Thus, the current modelled problem perfectly describes the physical problems of real world in such special circumstances.

## Introduction

Double-diffusive convection in flow has tremendous application, whereas, it is extensively used in many natural and scientific systems, in which the diffusion of heat and mass occur all together. The simultaneous diffusion of two quantities (temperature and concentration differences) are producing buoyancies, which generates the fluid motion. Thus this phenomenon is termed as double-diffusive convection in flow^1^. Buoyancies driven flows and double-diffusive convection in flows have been studied in many research papers, whereas, extensive problems have been solved of such kind, however, the gradients of both the diffusion variables are inducing the fluid motion and the gradients occur due to two different density gradients with different rates of diffusion^2^. This phenomenon frequently occurs in nature and many other industrial processes. The melting of ice and cooling of air near its surface, the intrusion of sea water into lakes and the crystallization process of magma intrusions in the crust of earth are the popular examples of natural systems of double-diffusive convection in flows. Moreover, the diffusion of heat and mass results in massive double-diffusive instabilities, whereas, this mechanism is called the “salt-fingers”^3,4^. The problems of double-diffusive convection in flows have extensive applications in industries and power plants of geothermal energy. These simulations have vast uses in the formation of chemical derivatives of natural gas and petroleum. The most important potential uses for double-diffusive convection in flow involves the manufacturing of foods in industries. Moreover, these studies are frequently used in grain and energy storages, transport of moisture in engineering systems, the formation of micro structures during the cooling of molten metals, fluid flows around shrouded heat-dissipation fins, diffusion of chemicals (solid and liquid fog and smoke particles) in soil (air) and cleaning and dyeing process. The oxidation of metallic surfaces and solidification of different equipment (devices) are usually carried out by using the phenomenon of double-diffusive convection in flows.

In this connection, the earliest investigations of Somers^5^ are very famous and the study has clearly described the mechanism of double-diffusive convection in fluid flow. The laminar flow of fluid develops under the influence of gravity and density gradients, whereas, the two diffused quantities and the simultaneous occurrence of these mechanisms usually caused such motion of fluids^6^. Note that this investigation has been carried out for flows along a vertical sheet and in plumes. Moreover, the double-diffusive convection in flow has been studied in^7–12^. Furthermore^13–16^, analyzed double-diffusive convection in flow inside a porous medium over a horizontal, inclined, and vertical surfaces. The effects of radiation on double-diffusive convection in flow over a cone is seen in^17^. The diffusion of heat and mass in a natural convection flow of a viscous and ideal fluid have been studied in^18^. Note that the MHD flow is maintained over truncated cone in the presence of radiation effects. More realistic investigations have been presented in^19^ and a model problem of double diffusive convection in incompressible and viscous fluid flows over a cone has been solved in the presence of cross diffusion. Numerical solutions of a double-diffusive convection flow inside a rectangular chamber (enclosure) with augmented/assisting and opposing diffusions gradients are presented in^20,21^. These solutions were crossed checked and compared with the experimental data, whereas, excellent agreement between the two is found. Moreover, multiple solutions of double diffusive convection in flow inside a vertical enclosure were presented in^22^.

Experimentation shows that, although, the diffusion of heat and mass in flow occurs at the same time, however, the connection between the fluxes and buoyancies complicated phenomenon, whereas, their analysis is the essential components of such investigations. Note that gradients of the both diffusion variables generate diffusions fluxes zones. Dufour or diffusion therm effect are caused by energy flux due to concentration gradient. On the other hand, in most cases, the mass fluxes have been created due to temperature gradient and this prolongs the Soret or thermal diffusion contributions. The combined Dufour and Soret effects are called the cross-diffusion phenomenon. The cross diffusion phenomenon is widely investigated in ideal fluids, whereas, experimental and theoretical consequences of Soret are analyzed for viscous fluids^23^.

In the present analysis we have studied the double-diffusive convection of heat and species mass in flow inside a heated, inclined and rectangular walls of plane geometry. The walls of the channels are located at $y=mx+h_{0}$, *m* ($-m$) is representing the slope of the upper (lower) wall of the channel and $h_{0}$ is its exit (inlet) when $x=0$ for converging (diverging) flow. The walls of the channel are heated/cooled with non-uniform temperature $T_{w}(x)$ and species concentration $C_{w}(x)$. The field variables are controlled by four PDEs and the relevant boundary conditions, however, the system of fundamental equations is taken in Cartesian Coordinates. Proper transformations are employed which reduce the PDEs into ODEs. The ODEs are solved with different methods/techniques. The system of ODEs involves several parameters such as the Reynolds number (*Re*), the slope (*m*), Prandtl number (*Pr*), Schmidt number (*Sc*), thermal ($Gr_{T}$) and solutal ($Gr_{S}$) Grashof numbers. The final system of equations is solved with Perturbation technique for a set of small values of parameter *m*, however, a numerical scheme is also employed to investigate the solution of the system of ODEs and the field variables are calculated accurately with the help of this technique for a wide range of the governing parameters. Note that the Perturbation method is only valid for small values of the governing parameters. Furthermore, a complete and an accurate numerical solution of the modeled problem is also given. The profiles of velocity and two diffusion variables are presented against the similarity variable for assisting and opposing flows. Moreover, skin friction, rates of the diffusing quantities are investigated for all cases of assisting and opposing flows in converging and diverging channels. Besides that, new variables are formed which relate the previous work of the same nature in Polar Coordinates to the new simulation in Cartesian Coordinates. The new simulations are compared with the classical work of Millsaps and Pohlhausen presented in White^24^, Laila et al.^25^ and Marwat et al.^26^, whereas, these results have been retrieved exactly. It is hoped that the present simulations perfectly narrates real world problems, frequently used in such special circumstances.

## Formulation of the problem

**Figure 1:**
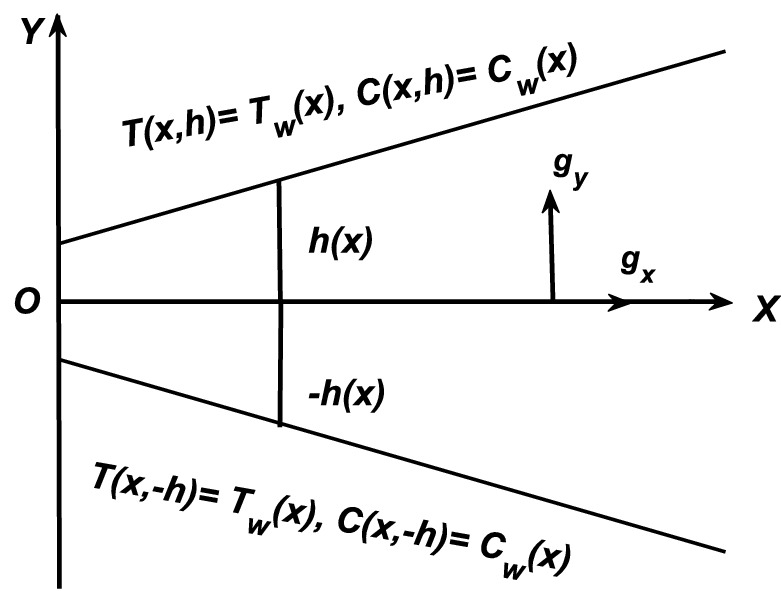
The geometry of the flow problem under consideration.

Double-diffusive convection in flow inside a converging and diverging channels of heated and rectangular walls is assumed in this analysis. Note that two dimensional flow of an incompressible viscous fluid is maintained in such channel (see Fig. 1). The heated walls of the inclined channel have variable temperature and species concentration. Moreover, the buoyancy forces are of significant order and they have been taken into consideration to simulate the flow problem. Note that we assumed the two dimensional form of governing equations in rectangular coordinates system. Besides that the fluid has uniform viscous, thermal and mass diffusivities. In view of these assumptions and approximations, we have the following set of equations:

1 $$\begin{aligned} \frac{\partial u}{\partial x}+ \frac{\partial v}{\partial y}= & {} 0, \end{aligned}$$

2 $$\begin{aligned} u\frac{\partial u}{\partial x}+v\frac{\partial u}{\partial y}= & {} -\frac{1}{\rho } \frac{\partial p}{\partial x}+g_{x}\beta (T-T_{0})+g_{x}\beta ^{*}(C-C_{0})+\nu \Big(\frac{\partial ^2 u}{\partial x^2}+\frac{\partial ^2 u}{\partial y^2}\Big), \end{aligned}$$

3 $$\begin{aligned} u\frac{\partial v}{\partial x}+v\frac{\partial v}{\partial y}= & {} -\frac{1}{\rho } \frac{\partial p}{\partial y}+g_{y}\beta (T-T_{0})+g_{y}\beta ^{*}(C-C_{0})+\nu \Big(\frac{\partial ^2 v}{\partial x^2}+\frac{\partial ^2 v}{\partial y^2}\Big), \end{aligned}$$

4 $$\begin{aligned} u\frac{\partial T}{\partial x}+v\frac{\partial T}{\partial y}= & {} \alpha \Big(\frac{\partial ^2 T}{\partial x^2}+\frac{\partial ^2 T}{\partial y^2}\Big), \end{aligned}$$

5 $$\begin{aligned} u\frac{\partial C}{\partial x}+ v\frac{\partial C}{\partial y}= & {} D_{b}\Big(\frac{\partial ^2 C}{\partial x^2}+\frac{\partial ^2 C}{\partial y^2}\Big), \end{aligned}$$

where Eqs. (1–3) are representing the continuity, *x* and *y* momentum equations, respectively, while Eqs. (4 and 5) are representing the well known energy and concentration equations. Note that velocity vector has two components i.e. *u* and *v* in the direction of *x* and *y*, respectively and *T*, *C*, *p* are the temperature, concentration and pressure variables, respectively. Moreover, $\rho$, $\nu =\frac{\mu }{\rho }$, $\alpha$, $D_{b}$, $\beta$ and $\beta ^{*}$ are density, viscous, thermal and mass diffusivities, co-efficient of thermal expansion and volumetric coefficient of mass transfer, whereas, $c_{p}, k, T_{0}$ and $C_{0}$ are specific heat, thermal conductivity, uniform temperature and concentration of the variable free stream at the centre of the channel, respectively. All these thermal properties are uniform through out the flow domain. Moreover, $g_{x}$, $g_{y}$ are components of acceleration due to gravity. Note that the viscous dissipation term is not taken into account in the energy Eq. (4), whereas, wall temperature $T_{w}(x)$ is greater than the temperature $T_{0}$ of free stream, developed at the centre of the channel and same is the case with the concentration function (*C*). The classical Stokes stream function may exist for such type of two dimensional flows. On the basis of this fact, a stream function ($\psi$) is introduced in term of axial and normal components of velocity i.e., *u* and *v* are defined by:

6 $$\begin{aligned} u=\frac{\partial \psi }{\partial y}, \quad and \quad v=-\frac{\partial \psi }{\partial x}. \end{aligned}$$

The vorticity equation is obtained by eliminating the pressure term between Eqs. (2 and 3) as:

7 $$\begin{aligned} \frac{\partial \omega }{\partial t}+ u\frac{\partial \omega }{\partial x}+v\frac{\partial \omega }{\partial y} = -g_{x}\beta \frac{\partial T}{\partial y} -g_{x}\beta ^{*}\frac{\partial C}{\partial y} + g_{y}\beta \Big(\frac{\partial T}{\partial x} -\frac{\partial T_{0}}{\partial x})+g_{y}\beta ^{*} (\frac{\partial C}{\partial x}-\frac{\partial C_{0}}{\partial x}\Big)+v\Big(\frac{\partial ^2 \omega }{\partial x^2}+\frac{\partial ^2\omega }{\partial y^2}\Big), \end{aligned}$$

where the vorticity function ($\omega$) for two dimensional flow is defined as:

8 $$\begin{aligned} \omega = \frac{\partial v}{\partial x}- \frac{\partial u}{\partial y}. \end{aligned}$$

The boundary conditions are specified at the upper (lower) wall and at the centre of the channel. The boundary conditions determine the value of field variable at these two locations. The converging (diverging) channel under consideration has symmetrical shape. The field variables have gotten fixed value at the upper boundary and centre of the channel in view of some known facts, whereas, they are known as no slip, symmetry conditions, no temperature and no concentration jump conditions for the field variables. The conditions, imposed at wall and centre of the converging (diverging) channel, are given below:

9 $$\begin{aligned} \begin{aligned} u(x,y)&=U(x), \quad \omega (x,y)=0, \quad \psi (x,y)=0, \quad T(x,y) = T_{0}, \quad C(x,y) = C_{0} \quad at \quad y=0,\\ u(x,y)&=0,\quad T(x,y)=T_{w}(x), \quad C(x,y)=C_{w}(x) \quad at \quad y=h(x). \end{aligned} \end{aligned}$$

Note that a variable free stream *U*(*x*) is developed at the centre of the channel. The quantities $T_{0}$ and $C_{0}$ are denoting the uniform temperature and constant species concentration of the free stream, whereas, $T_{w}(x)$ and $C_{w}(x)$ are variable temperature and concentration functions at the wall, respectively. In this stage, we introduced new functions $f(\eta )$, $\theta (\eta )$, $\phi (\eta )$ and $P(\eta )$ for the stream function $(\psi )$, temperature (*T*), concentration (*C*) and pressure (*p*) variables as:

10 $$\begin{aligned} \begin{aligned} \psi&=h(x)U(x)f(\eta ),\quad \theta (\eta )=\frac{T-T_{0}}{T_{w}(x)-T_{0}}, \quad \phi (\eta )=\frac{C-C_{0}}{C_{w}(x)-C_{0}}, \\ P(\eta )&= \frac{h^2(x)p}{\nu \rho h_{0} U_{0}} \ \ where, \quad \eta =\frac{y}{h(x)}, \end{aligned} \end{aligned}$$

where, $T_{w}(x)=T_{0}+\frac{T_{1} h_{0}^3}{(mx+h_{0})^3}$, $C_{w}(x)=C_{0}+\frac{C_{1}h_{0}^3}{(mx+h_{0})^3}$, and $U(x)=\frac{U_{0}}{m\chi +1}$. Note that *U*(*x*) is free stream at the center of channel, $T_{1}(C_{1})$ is the controlling parameter for variable wall’s temperature (concentration), $h(x)=mx+h_{0}$ and $\chi =\frac{x}{h_{0}}$ while $m(-m)$ is the slope of the channel’s upper wall (lower) wall, $h_{0}$ is the channel half spacing when $x=0$. Invoking the definition of stream function $(\psi )$ provided in Eq. (10) into Eqs. (7 and 8) and the vorticity equation is transformed into the following ODE:

11 $$\begin{aligned} \begin{aligned}{}&(1+m^2\eta ^2)^2 f^{(iv)}+ 12 m^2\eta (1+m^2\eta ^2)f'''(\eta )+ 12 m^2(1+3m^2\eta ^2)f''(\eta )\\&+ 2m Re(1+m^2\eta ^2)f''(\eta )f'(\eta ) +4m^3 Re\eta f'^{2}(\eta ) +24 m^4\eta f'(\eta )+(m\eta Gry_{T}+Grx_{T})\theta '(\eta )\\&+3m Gry_{T}\theta (\eta )+(m\eta Gry_{S}+Grx_{S})\phi '(\eta )+3m Gry_{S}\phi (\eta )=0. \end{aligned} \end{aligned}$$

The energy and concentration equations i.e. Eqs. (4 and 5) are converted into the following ODEs after the use of the transformation provided in Eq. (10).

12 $$\begin{aligned}{}&(1+m^2\eta ^2)\theta ''(\eta )+8m^2\eta \theta '(\eta )+3 m Pr Re \theta (\eta )f'(\eta )+12 m^2 \theta (\eta )=0, \end{aligned}$$

13 $$\begin{aligned}{}&(1+m^2\eta ^2)\phi ''(\eta )+8m^2\eta \phi '(\eta )+3 m Re Sc \phi (\eta )f'(\eta )+12 m^2 \phi (\eta )=0. \end{aligned}$$

where prime represents the differentiation with respect to $\eta$. Moreover, the dimensionless numbers are defined by:

$$\begin{aligned} Re= & {} \frac{h_{0}U_{0}}{\nu },\ \ Grx_{T}=\frac{g_{x}\beta T_{1} h_{0}^3}{\nu ^2},\ \ Gry_{T}=\frac{g_{y}\beta T_{1} h_{0}^3}{\nu ^2}, \\ Grx_{S}= & {} \frac{g_{x}\beta ^{*} C_{1} h_{0}^3}{\nu ^2}, \ \ Gry_{S}=\frac{g_{y}\beta ^{*} C_{1}h_{0}^3}{\nu ^2}, \ \ Pr=\frac{\mu c_{p}}{k} \ \ and \ \ Sc=\frac{\nu }{D_{b}}. \end{aligned}$$

The above dimensionless numbers are known in the literature as Reynolds number (*Re*), modified thermal Grashof numbers ($Grx_{T}, Gry_{T}$), modified solutal Grashof numbers $(Grx_{S}, Gry_{S}$), Prandtl number (*Pr*) and Schmidt number (*Sc*). Note that $g_{x}=g\sin \gamma , g_{y}=g\cos \gamma$, where, $\gamma =\tan ^{-1}m$ i.e. $\gamma$ is the inclination angle of the wall and for $\gamma =\frac{\pi }{2}$, we obtained that $g_{y}=0 \ \  \&  \ \ g_{x}=g$ and for this choice of $\gamma$, we get the flow model of double-diffusive convection of heat and species mass in viscous flow inside a vertical channel of parallel walls.

The boundary conditions in Eq. (9) for *f*, $\theta$ and $\phi$ are now transformed into the following exact form:

14 $$\begin{aligned} f(0)= & {} 0, \ \ f''(0)=0, \ \ f'(0)=1, \ \ f'(1)=0, \end{aligned}$$

15 $$\begin{aligned} \theta (0)= & {} 0,\ \ \theta (1)=1, \end{aligned}$$

16 $$\begin{aligned} \phi (0)= & {} 0,\ \ \phi (1)=1. \end{aligned}$$

## Evaluation of pressure

The pressure term is simply obtained from Eqs. (2 and 3) when we substituted the transformation from Eq. (10) into Eqs. (2 and 3). So in view of Eq. (10), the Eq. (2) becomes:

17 $$\begin{aligned} \begin{aligned}{}&m \eta P'(\eta ) +2m P(\eta )+(1+m^2\eta ^2)f'''(\eta )+4m^2\eta f''(\eta ) \\&+m Re f'^2(\eta )+2m^2f'(\eta )+Grx_{T}\theta (\eta )+Grx_{S}\phi (\eta )=0. \end{aligned} \end{aligned}$$

Similarly, the transformations for *p*, *u*, *v*, *T*, *C*,  and $\eta$ are substituted into Eq. (3) and we get:

18 $$\begin{aligned} \begin{aligned}{}&P'(\eta )-m\eta (1+m^2\eta ^2)f'''(\eta ) -2m(1+3m^2\eta ^2)f''(\eta ) -m^2 Re\eta f'^2(\eta ) \\&-6m^3\eta f'(\eta )-Gry_{T}\theta (\eta )-Gry_{S}\phi (\eta )=0. \end{aligned} \end{aligned}$$

Note that Eq. (17) contains $P(\eta )$ and $P'(\eta )$ (representative of pressure *p*), whereas, Eq. (18) contains $P'(\eta )$ only. After solving these two equations simultaneously and the pressure term is obtained as:

19 $$\begin{aligned} \begin{aligned}{}&P(\eta )=\frac{-1}{2m}[(1+ m^2 \eta ^2)^2 f'''(\eta )+ 6m^2\eta (1+m^2\eta ^2)f''(\eta )+m Re(1+m^2\eta ^2)f'^2(\eta )\\&+2m^2(1+3m^2 \eta ^2 ) f'(\eta )+(Grx_{T}+ m\eta Gry_{T})\theta (\eta )+(Grx_{S}+m \eta Gry_{S})\phi (\eta )]. \end{aligned} \end{aligned}$$

Note that Eq. (19) contains $P(\eta )$ (the representative of pressure term), *f*, derivatives of *f*, $\theta , \phi$, similarity variable $\eta$, *m*, *Re*,  two components of each *Gr*. So $P(\eta )$ can be easily determined by substituting value of $f, \theta$ and $\phi$ which can be determined from the solution of Eqs. (11–13) with boundary conditions in Eqs. (14–16) either numerically (*bvp*4*c* solution) or analytically (perturbation and closed form solution). Moreover, Eq. (19) shows that the pressure $P(\eta )$ term will strictly vary with $m, \eta , Re, Gr_{T}$ and $Gr_{S}$.

**Figure 2:**
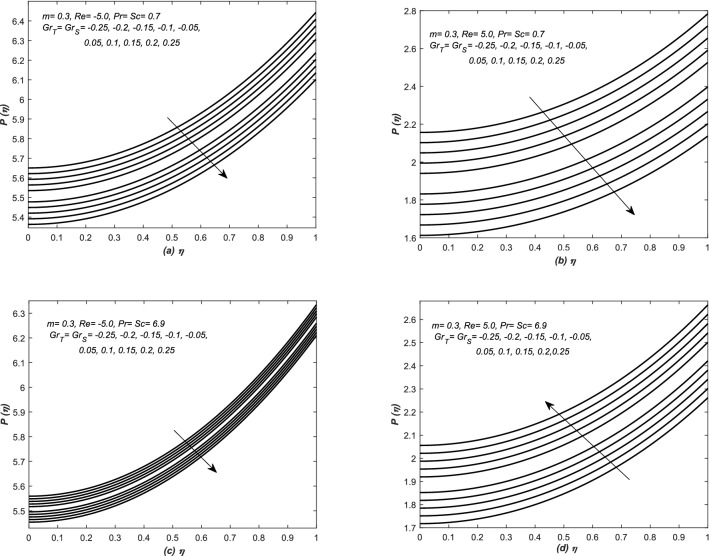
The dimensionless pressure or $P(\eta )$ is plotted against $\eta$ for various values of thermal and solutal Grashof numbers such that $Gr_{T}=Gr_{S}$. Note that these investigations are carried out for the flow of air and water in converging and diverging channels.

In Fig. 2, the dimensionless pressure $P(\eta )$ is plotted against $\eta$ for various values of *Gr* (both thermal and solutal), whereas, the experiments have been carried out for both converging and diverging flows. In all these subplots, the pressure has been risen against the similarity variable $\eta$ and shows non-linear behaviour, however, it is increasing function of $\eta$ and decreased with the increasing values of *Gr* (both thermal and solutal) for flow of water and air in a converging channel. In case of diverging flow of air (water), the pressure is decreased (increased) with the increasing of *Gr* (both thermal and solutal). In diverging flow, an abrupt change in the profile of pressure distribution is observed for any small variation in *Gr* (both thermal and solutal), however, in case of converging flow, slight variation in the profiles of pressure distribution $P(\eta )$ is observed against *Gr* (both thermal and solutal). Note that we did not consider and present the case of favourable pressure. However, in such situations the observations are totally opposite to that.

      Next, we evaluate the pressure at top wall of the channel. For this, we substitute $\eta =1$ in Eq. (19), and use the B.Cs in Eqs. (14–16), we obtained that:

20 $$\begin{aligned} P(1)= \frac{-1}{2m}[(1+ m^2)^2 A_{1} + 6m^2(1+m^2) A_{2}+Grx_{T} +Grx_{S}+ m(Gry_{T}+Gry_{S})], \end{aligned}$$

where $A_{1}= f'''(1)$ and $A_{2}= f''(1)$. This analysis helped us to classify the cases of favourable and adverse pressures at the top walls of the channel.

**Figure 3:**
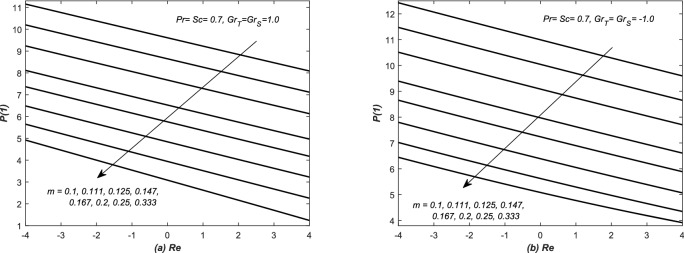
The dimensionless pressure at wall i.e. *P*(1) is plotted against the Reynolds number *Re*, whereas, assisting and opposing flows of air are taken in inclined for different values of slope *m*.

**Figure 4:**
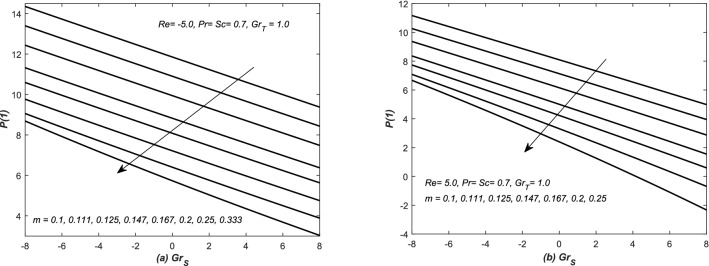
The dimensionless pressure at wall i.e. *P*(1) is plotted against solutal Grashof numbers $Gr_{S}$, whereas, flow of air is taken in (**a**) converging and (**b**) diverging channels.

In Fig. 3, the pressure at the upper wall i.e. *P*(1) is evaluated and plotted against *Re* for both assisting and opposing flows in inclined channel. It is observed that the pressure at the upper wall is decreased linearly against *Re* for increasing values of *m*. In Fig. 4, the pressure at the upper wall i.e. *P*(1) is evaluated against the Grashof number *Gr* for both converging and diverging flows. The pressure at the upper wall is decreased linearly against *Gr* in these situations for the increasing values of *m*. Note that fluid exerts more pressure at the wall in case of converging flow (observation from Figs. 3 and 4).

## Perturbation solution for small *m*

In this section we found and analyzed the perturbation solutions of Eqs. (11–16). The system of equations is solved with the help of well established and standard technique i.e. perturbation method for small value of the parameter *m*, however, its solution is also presented by an accurate numerical method. Note that the perturbation method requires a parameter in the problem, although, the system of equations contains eight different parameters, however, the inclination parameter happens to be a small quantity in many engineering system, which are concerned with the diffusion of heat and mass in flow, therefore, a regular perturbation technique has been taken into account for solving the final problem. Effects of different parameters have been presented on the profiles of field quantities, skin friction and rate of two diffusions at the top surface of the channel, however, they have been discussed numerically. This perturbation technique provides accurate solutions for small value of the parameters. It is assumed that *m* (the slope of the upper wall) is a small quantity and we need to determine the perturbation solution of the problem for small values of *m*. In order to find the perturbation solution for small *m*, the method requires to write the unknown functions *f*, $\theta$ and $\phi$ in the form of infinite series. Here we express unknown quantities *f*, $\theta$ and $\phi$ in terms of small quantity *m* as follows:

21 $$\begin{aligned} f(\eta )= & {} f_{0}(\eta )+m f_{1}(\eta )+O(m^2), \end{aligned}$$

22 $$\begin{aligned} \theta (\eta )= & {} \theta _{0}(\eta )+m\theta _{1}(\eta )+O(m^2), \end{aligned}$$

23 $$\begin{aligned} \phi (\eta )= & {} \phi _{0}(\eta )+m\phi _{1}(\eta )+O(m^2). \end{aligned}$$

The series (for *f*, $\theta$ and $\phi$ in the above equations) are substituted in the system of ODEs in Eqs. (11–13) and B.Cs in Eqs. (14–16). After that, the coefficients of *m* are equated on both sides of these equations and finally, we get two systems of three ODEs with the relevant boundary conditions. These two systems contain the unknown functions $f_{0}, f_{1}, \theta _{0}, \theta _{1}, \phi _{0}$ and $\phi _{1}$, eventually, they are determined from the solution of systems of BVPs which are given below:

24 $$\begin{aligned} f_{0}= &\; {} \frac{1}{72}(72\eta +a_{0}\eta ^3+a_{1}\eta ^4), \theta _{0}=\eta , \phi _{0}=\eta , \end{aligned}$$

25 $$\begin{aligned} f_{1}= &\; {} b_{0}\eta ^3+b_{1}\eta ^4+b_{2}\eta ^5+b_{3}\eta ^6+b_{4}\eta ^7+b_{5}\eta ^8+b_{6}\eta ^9, \end{aligned}$$

26 $$\begin{aligned} \theta _{1}= &\; {} \frac{1}{120}({c_{0}\eta +c_{1}\eta ^3+c_{2}\eta ^5+c_{3}\eta ^6}), \end{aligned}$$

27 $$\begin{aligned} \phi _{1}= &\; {} \frac{1}{120}(d_{0}\eta +d_{1}\eta ^3+d_{2}\eta ^3+d_{3}\eta ^5+d_{0}\eta ^6). \end{aligned}$$

Note that the different coefficients of the polynomials, appeared in Eqs. (24–27), are given below:

$$\begin{aligned} a_{0}= &\; {} 4(Grx_{S}+Grx_{T}-6) ; a_{1}=-3(Grx_{S}+Grx_{T});\\ b_{0}= & \;{} Re\Big(\frac{a_{0}}{432}+\frac{a_{0}^2}{51840}+\frac{a_{1}}{540}+ \frac{a_{0} a_{1} }{27216}+\frac{a_{1}^2}{54432}\Big)+ Grx_{S}\Big(\frac{d_{0}}{2160}+\frac{d_{1}}{7200}+\frac{d_{2}}{15120}+\frac{d_{3}}{20160}\Big)\\&\qquad +Grx_{T}\Big(\frac{c_{0}}{2160}+\frac{c_{1}}{7200}+\frac{c_{2}}{15120}+\frac{c_{3}}{20160}\Big)+\frac{Gry_{S}}{18}+\frac{Gry_{T}}{18};\\ b_{1}= & \;{} -\frac{-1}{2880}(d_{0}Grx_{S}+c_{0}Grx_{T}); b_{2}=-\Big(\frac{a_{0}Re}{720}+\frac{Gry_{S}}{30}+\frac{Gry_{T}}{30}\Big);\\ b_{3}= & \;{} -\Big(\frac{a_{1}Re}{1080}+\frac{d_{1}Grx_{S}}{14400}+ \frac{c_{1}Grx_{T}}{14400}\Big); b_{4}=-\frac{a_{0}^2Re}{120960}; \ \ b_{5}=-\Big(\frac{a_{0}a_{1}Re}{72576}+\frac{d_{2}Grx_{S}}{40320}+ \frac{c_{2}Grx_{T}}{40320}\Big);\\ b_{6}= & \;{} -\Big(\frac{a_{1}^2Re}{163296}+\frac{d_{3}Grx_{S}}{60480}+ \frac{c_{3}Grx_{T}}{60480}\Big); c_{0}=PrRe(42+Grx_{S}+Grx_{T}); \\ c_{1}= & \;{} -60PrRe; \ \ c_{2}=3PrRe(6-Grx_{S}-Grx_{T}); c_{3}=2ReSc(Grx_{S}+Grx_{T}); \\ d_{0}= & \;{} ScRe(42+Grx_{S}+Grx_{T}); d_{1}=-60ScRe; \ \ d_{2}=3ScRe(6-Grx_{S}-Grx_{T});\\ \end{aligned}$$

and $d_{3}=2ReSc(Grx_{S}+Grx_{T}).$. The final form of the series/perturbation solution can be achieved by placing the expression for $f_{0}$, $f_{1}$, $\theta _{0}$, $\theta _{1}$, and $\phi _{0}$ and $\phi _{1}$ from Eqs. (24–27) into Eqs. (21–23). It is important to note that this perturbation result works only for small values of *m* and other physical parameters appeared in the modeled equations.

## Comparison of present simulation & its numerical solutions with published work and analytical results

**Figure 5:**
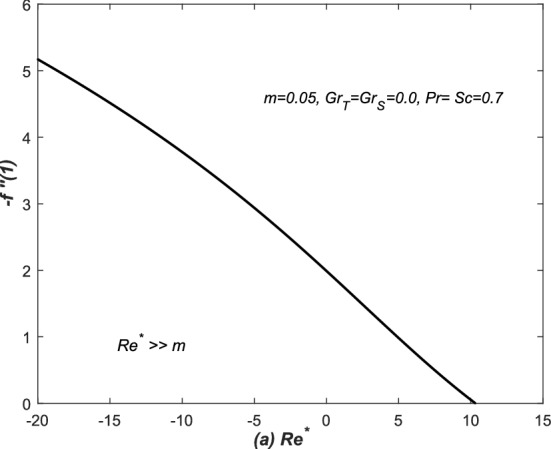
The skin friction coefficient ($-f''(1)$) is plotted against modified Reynolds number $Re^{*}=m Re$ for $m<<Re^{*}$.

**Figure 6:**
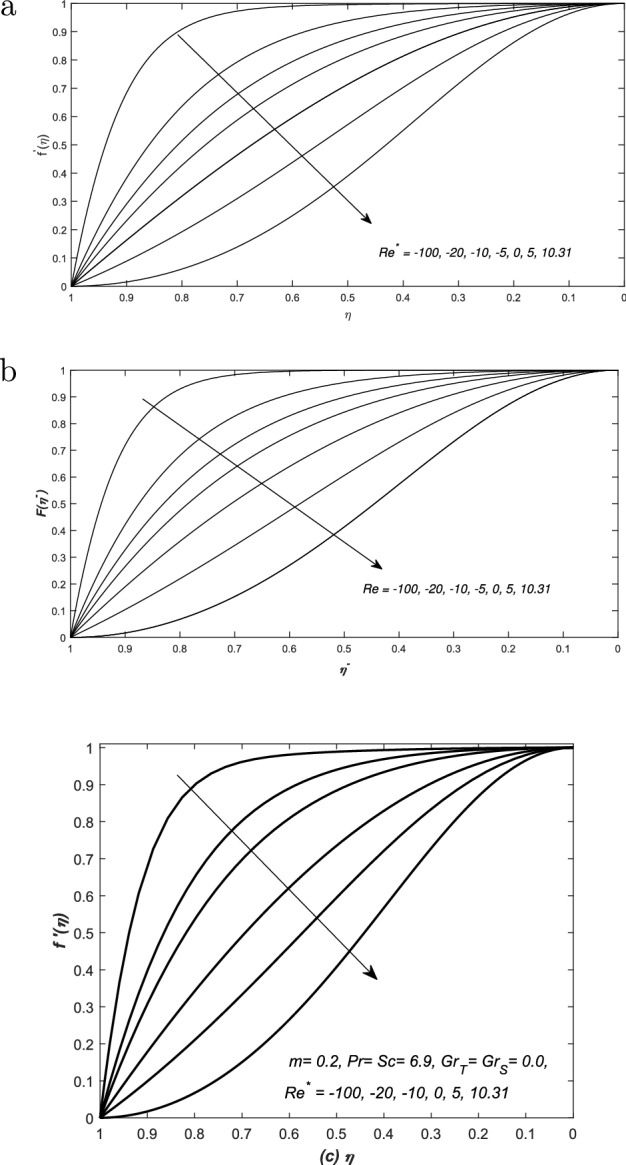
The axial velocity $f'(\eta )$ is plotted against the similarity variable $\eta$ for different values of modified Reynolds number $Re^{*}=mRe$ and the results are compared with published work of (**a**) Laila et al.^25^ and (**b**) Marwat et al.^26^. The profiles in (**c**) showed that curves of $f'(\eta )$ are exactly matched with these two published papers. Note that the profiles in (**a**) and (**b**) are published in the Fig. 4 and 16 of R. Laila et al.^25^ and Marwat et al.^26^, respectively.

We tried to compare the present investigations with the classical analysis of flow in a converging and diverging channels and retrieved the published results from the current modeled problem, therefore, the numerical solutions of present simulations are juxtaposed against the classical work. The skin friction coefficient is evaluated at the upper wall of the channel and its final form is represented by $-f''(1)$ in Eq. (28), whereas, $-f''(1)$ is plotted against the modified Reynold’s number $Re^{*}=m Re$ where $m=o(Re)$ in Fig. 5. The profile is exactly matched with the previously published profile of Millsaps and Pohlhausen, discussed in White^24^. He claimed that the profile of skin friction against modified Reynold’s number $Re^{*}$ is relatively closed to a straight line for negative values of $Re^{*}$. Whereas, for $Re^{*}>0$, the profile of $-f''(1)$ gets zero value at $Re^{*}\simeq 10.31$. Note that these authenticated remarks of Millsaps and Pohlhausen are retrieved exactly from Fig. 5 of the present modeled problem. Moreover, solutions of the new modeled problems are also compared with two latest published papers and the results are exactly agreed with the profiles of these two published articles. The dimensionless velocity i.e. $f'(\eta )$ is plotted against the similarity variable $\eta$ in Fig. 6 for different values of $Re^{*}=m Re$ and $m=0.2$. The profiles are obtained by solving the system of Eqs. (11–14). We exactly found the same observations as reported in Laila et al.^25^ and Marwat et al.^26^.

The analytical solution of Eqs. (21–23) has been found by using the perturbation method and presented in Eqs. (24–27). The numerical solution of Eqs. (11–14) is compared with this analytical solution and the results are shown in Table 1. The unknown functions *f*, $\theta$ & $\phi$ are evaluated at some 41 mesh points between 0 and 1, whereas, all these values are not listed in this table and only selected values of $\eta$ and that of the unknown functions have been mentioned in the table. The different parameters value are taken such that $m = 0.1,Re = -2.0, Pr=Sc=0.7, Gr_{T}=Gr_{S}=0.5$. It has been noted from this table that in most cases, the two solutions are exactly same up to four decimal places, whereas, in the remaining cases, a minimum difference between the two solutions has been observed. As a result, the two solutions are exactly matched with each other.

**Table 1:** The comparison of approximate analytical solution in Eqs. (21–23) with numerical solutions of the unknown functions in Eqs. (11–14) where the subscripts *n* and *a* of the variables $f,\theta ,\phi$ are used for numerical and approximate analytical solutions, respectively, whereas, $\Delta \theta =\theta _{n}-\theta _{a}$ & $\Delta \phi = \phi _{n}-\phi _{a}$.

$$\eta $$	$$f_{n} $$	$$f_{a}$$	$$f_{n}-f_{a}$$	$$f'_{n} $$	$$f'_{a}$$	$$f'_{n}-f'_{a}$$	$$\theta _{n}= \phi _{n}$$	$$\theta _{a}=\phi _{a}$$	$$\Delta \theta = \Delta \phi $$
0.0000	0.0000	0.0000	0.0000	1.0000	1.0000	0.0000	0.0000	0.0000	0.0000
0.1531	0.1519	0.1519	0.0000	0.9774	0.9774	0.0000	0.1508	0.1510	0.0002
0.1837	0.1837	0.1837	0.0000	0.9674	0.9674	0.0000	0.1810	0.1812	0.0002
0.3367	0.3244	0.3244	0.0000	0.8900	0.8900	0.0000	0.3328	0.3330	0.0002
0.4898	0.4518	0.4518	0.0000	0.7659	0.7659	0.0000	0.4858	0.4861	0.0003
0.5510	0.4968	0.4967	0.0001	0.7030	0.7029	0.0001	0.5474	0.5477	0.0003
0.6122	0.5377	0.5376	0.0001	0.6323	0.6323	0.0000	0.6092	0.6094	0.0002
0.7143	0.5955	0.5955	0.0000	0.4970	0.4970	0.0000	0.7125	0.7127	0.0002
0.7959	0.6311	0.6311	0.0000	0.3729	0.3730	0.0001	0.7952	0.7953	0.0001
0.9286	0.6656	0.6656	0.0000	0.1408	0.1407	0.0001	0.9289	0.9289	0.0000
0.9796	0.6702	0.6702	0.0000	0.0414	0.0413	0.0001	0.9798	0.9798	0.0000
1.0000	0.6707	0.6707	0.0000	0.0000	0.0000	0.0000	1.0000	1.0000	0.0000

## Numerical results and their graphical discussion

Numerical solution of the systems of equations and boundary conditions in Eqs. (11–16) is found with the help of using *bvp*4*c* package of MATLAB and it is a finite difference method which depends on collocation polynomial. So this scheme evaluates the field variables *f*, $\theta$ and $\phi$ from the system of boundary value ODEs for fixed numerical value of the parameters.

### Assisting and opposing flows

**Figure 7:**
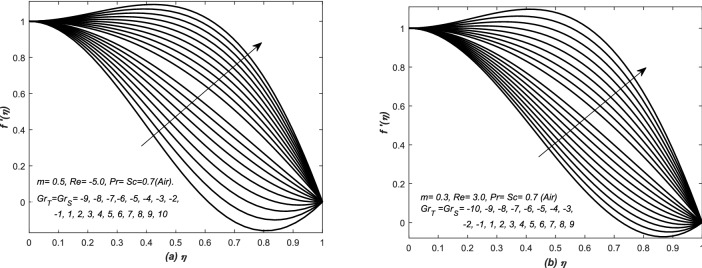
The dimensionless velocity $f'(\eta )$ is plotted against the similarity variable $\eta$ for assisting and opposing flow of air inside (**a**) converging and (**b**) diverging channels.

**Figure 8:**
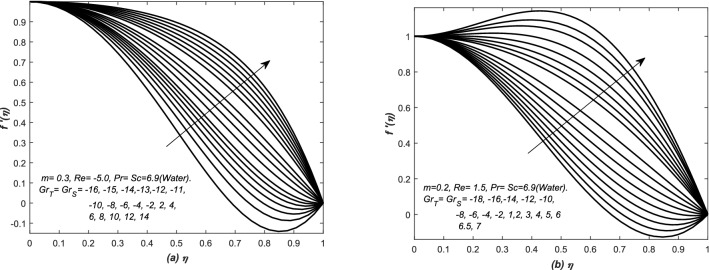
The dimensionless velocity $f'(\eta )$ is plotted against the similarity variable $\eta$ for assisting and opposing flow of water inside (**a**) converging and (**b**) diverging channels.

In Eq. (11), two parameters i.e. $Gr_{T}$ and $Gr_{S}$ are associated with diffusion buoyancies, respectively. The solutions in this section are categorized on the basis of positive and negative values of these two parameters i.e. thermal and solutal Grashof numbers. The profiles of axial velocity are plotted against the similarity variable $\eta$ in Figs. 7 and 8 for various values of both $Gr_{T}$ and $Gr_{S}$. The assisting and opposing flows are examined in converging and diverging channels in Figs. 7 and 8, respectively. There are three different plots in each figure and this categorization is based on the physical structure of the flow inside the channel of inclined walls $(i) Re<0, (ii) Re>0$. The analysis is extended for fluids of two different thermal and mass diffusivities. Therefore, two groups of profiles are presented in each graph of these figures. The upper profiles are corresponding to assisting flow, whereas, the lower group of profiles is representing the opposing flow patterns.

In Fig. 7, the velocity curves are graphed for various values (negative and positive) of $Gr_{T}= Gr_{S}$, whereas, flow of air is undertaken in both converging and diverging channels. For a fixed value of parameters, the velocity curves increased with the increasing value of $Gr_{T}= Gr_{S}$, however, for increasing value of these parameters, a peak is seen in the velocity curves of Fig. 7 and this case is called an assisting flow. The variation in velocity curves is more quick in Fig. 7a as compared to Fig. 7b. Moreover, the profiles exceeded the maximum limit of 1 in case of assisting flows, whereas, it also crossed the minimum value 0 (no-slip condition at top wall) due to opposing flow (see Fig. 7(a,b) and an increase in the height of peaks has been observed for large $Gr_{T}= Gr_{S}$. Moreover, in case of strong assisting (opposing) flow, the boundary layer behavior of velocity profile is depicted near the centre (wall) of the both converging and diverging channels. Similarly, the cases of assisting and opposing flows are also investigated in Fig. 8 for the flow of water inside converging and diverging channels. Two sets of profiles are shown in each graphs of Fig. 8, the upper set of profiles is corresponding to assisting flow, whereas, the lower one is representing the opposing flow. In Fig. 8(a,b), the velocity profiles are graphed for different positive and negative values of $Gr_{T}=Gr_{S}$. For that choice of parameters value, abrupt changes have been noted in the profiles of velocity and it increases with the increasing value of $Gr_{T}= Gr_{S}>0$, moreover, for increasing values of these parameters, a peak can be seen in the velocity profiles and it is the clear cut situation of assisting flow, however, the peak in the profiles is more dominant in Fig. 8b. The profile exceeds the maximum limit (i.e. 1) of velocity in case of assisting flows, whereas, it also crosses the minimum value (i.e. 0) of velocity due to opposing flow. These two figures i.e. Figs. 7 and 8 are telling the prominent and significant roles of *Gr* (both thermal and solutal).

### Temperature and species concentration

**Figure 9:**
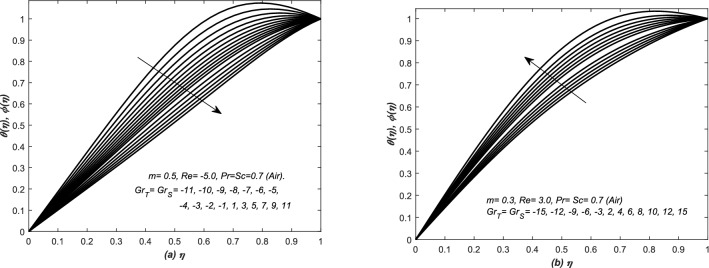
The dimensionless temperature $\theta (\eta )$ and species concentration $\phi (\eta )$ are plotted against the similarity variable $\eta$ for assisting and opposing flow of air inside (**a**) converging and (**b**) diverging channels.

**Figure 10:**
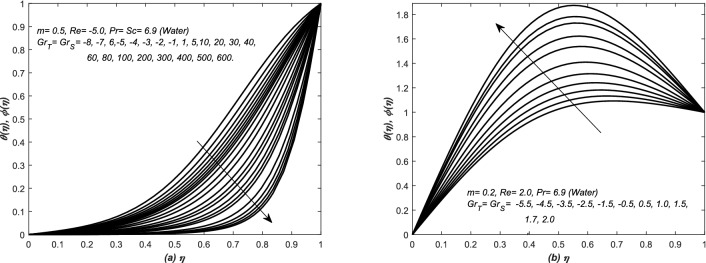
The dimensionless temperature $\theta (\eta )$ and species concentration $\phi (\eta )$ are plotted against the similarity variable $\eta$ for assisting and opposing flow of water inside (**a**) converging and (**b**) diverging channels.

In Figs. 9 and 10, the profiles of both the diffusion variables are plotted against $\eta$ for various and distinct values of $Gr_{T}=Gr_{S}$ and each line or curve in these graphs is showing the temperature and concentration distributions. In Figs. 9a and 10a, both the profiles of two diffusions distribution are graphed together and discussed for converging flow, whereas, in Figs. 9b and 10b diverging flow cases are examined. In Fig. 9, the temperature and concentration distributions are decreased (increased) uniformly with the increasing values of $Gr_{T}$ and $Gr_{S}$. The profiles of two diffusion variables are concave downward.

In Fig. 10a, the profiles of two diffusion distributions are decreased uniformly with the increasing values of $Gr_{T}$ and $Gr_{S}$. Diffusion boundary layers are observed in the surrounding of the centre of the channel, moreover, the boundary layers thickness remains dominant for large values of $Gr_{T}$ and $Gr_{S}$. In addition, the figure shows that the fluid is hotter and highly saturated near the centre for large values of $Gr_{T}$ and $Gr_{S}$, however, the profiles of asymptotic nature reach to a constant value 1. Moreover, the profiles of both these quantities have changed uniformly with the parameters, involved in the final system of equations. Note that the numerical value of the two diffusion functions, defined at wall, are greater than two diffusions functions, defined at the centre of the channel. The two diffusion variables are decreased gradually and smoothly near the wall, however, they are decreased suddenly to zero near the centre. In other words, for large values of $Gr_{T}$ and $Gr_{S}$, the temperature and species concentration of fluid suddenly rise at the centre of converging channel. In Fig. 10b, the temperature curves have peak, which is corresponding to the large value of $Gr_{T}$ and $Gr_{S}$. Note that all these profiles are concave downward. Moreover, in case of strong opposing flows, the boundary layer behavior of the profiles has seen near the upper wall for large $Gr_{T}= Gr_{S}$.

### Stream contours

**Figure 11:**
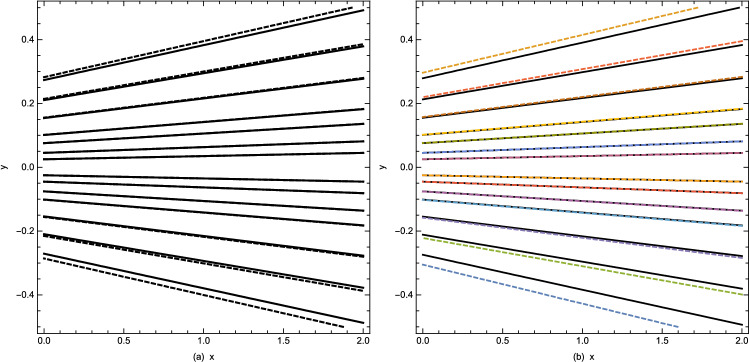
Stream contours are plotted for $$Gr_{T}= Gr_{S}=1.5$$ (solid lines) and $$Gr_{T}= Gr_{S}=-1.5$$ (dotted lines) inside (**a**) converging ($$Re=-5.0$$) (**b**) diverging ($$Re=-5.0$$) channels. While the other parameters are $$ m = 0.2, Pr = 1.0, Sc = 1.0$$.

In Fig. 11 the contours of stream lines for both assisting and opposing flows are plotted inside converging and diverging channels. These profiles are obtained from the perturbation solution in Eq. (21) for two different values of thermal and solutal Grashof numbers i.e. $Gr_{T}= Gr_{S}=\pm 1.5$. It is depicted from Fig. 11(a,b) that the thermal and solutal Grashof numbers have minor effects on the streamlines near the centre of the channel inside both converging and diverging flows. The profiles of stream lines slightly vary in converging flow. However, these variations are prominent in diverging flow. Furthermore, the behavior of streamlines is identical at the center of channel for the given set of parameters values.

Thermal and salinity gradients are involved in convection processes known as thermohaline convection, whereas, the marangoni convection is usually associated with the gradients of temperature and concentration at the surface. Sun is an important example of double-diffusive convection in natural setting as diffusion of temperature and gases at different rates on its surface. Contrary to convective flow problems, the simulations of double-diffusive convection in flow are more complicated and the main reason for this complexity is two way coupling between the velocity vector and the two diffusion variables. In double-diffusive convection, assisting and opposing flows are often discussed in the literature. In assisting flow, the two supporting buoyancies are prevailing in the direction of flow while in opposing flow, opposing buoyancies exert in anti-direction of flow. However, an accurate and standard relations have been established in this analysis to determine the combined effects of buoyancies or diffusion driven flow that would develop at the vicinity of an inclined wall of the converging/ diverging channel. A double-diffusive convection flow problem is solved with the help of asymptotic and numerical techniques and the analysis is extended for nonuniform diffusion variables at the sheet. The governing equations are upgraded and their modified version is formed with the help of well-known Boussinesq approximations, whereas, the new system is transformed into a set of equations in view of similarity variable for combined buoyancy effects. The nature and type of the similarity was not changed for such flow models, whereas, the classical similarities of a single buoyancy flow have not been under taken in this analysis. The governing equations were tested for flow of air and water in such circumstances, whereas, they are simplified and solved, however, the results are evaluated for multiple values of Prandtl and Schmidt numbers in case of both assisting and opposing flows. Moreover, effects of existing parameters have been seen on the velocity profiles and the two diffusion variables and the stability of laminar flow has been checked for a wide range of parameters value. The obtained results were analyzed and compared with the solutions found by different methods, however, the asymptotic solutions have certain limitations and the possible reason for the failure of these approximate solutions is genuine. It happened due to double diffusion or buoyancies or two way coupling of governing equations and weights of parameters associated with these forces terms.

### Evaluation of skin friction and rates of the two diffusion coefficients

The effectiveness of flow and the two diffusion coefficients have been determined and measured here. This task is accomplished when we calculate the skin friction and the rates of two diffusion functions, at the surface of inclined wall. In order to evaluate the skin friction $C_{f}$, we bring into consideration the definition of shear stress $\tau =\mu \Big(\frac{\partial u}{\partial y}+ \frac{\partial v}{\partial x}\Big)$ and find it at wall $\tau _{w}=\tau |_{y=h}=-\frac{(-1+m^2)\mu h_{0}U_{0}f''(1)}{h^2}$. After the non-dimensionalization of shear stress at wall $(\tau _{w})$ by $\frac{(-1+m^2)\mu h_{0}U_{0}}{h^2}$, we obtained that:

28 $$\begin{aligned} C_{f}= \frac{h^2 \tau _{w}}{(-1+m^2)\mu h_{0}U_{0}}=-f''(1). \end{aligned}$$

Similarly, the heat flux or the rate of heat transfer is defined by the Fourier’s Law of heat conduction,

$$\begin{aligned} {\mathbf {q}}= -k\nabla T= -k\Big(\frac{\partial T}{\partial x}, \frac{\partial T}{\partial y}\Big) = -\frac{k T_{0}}{h^4}[-m(3 \theta (\eta )+\eta \theta '(\eta )), \theta '(\eta )], \end{aligned}$$

so the rate of heat transfer at wall $(\mathbf {q_{w}})$ in view of Eqs. (10) becomes:

29 $$\begin{aligned} \mathbf {q_{w}}= -k \nabla T |_{y=h}=-\frac{k T_{0}}{h^4}(-m(3+ \theta '(1)), \theta '(1)). \end{aligned}$$

On the other hand, the heat transfer coefficient is defined by $\zeta =\frac{q_{w}h^3}{T_{0}}$. The dimensionless form of heat transfer coefficient $(\zeta )$ gives the Nusselt number $(Nu_{h})$ and we get:

30 $$\begin{aligned} Nu_{h}=\frac{\zeta h}{k}=\frac{k}{h}[-m(3+ \theta '(1)), \theta '(1)]. \end{aligned}$$

Note that in either case, the Nusselt number ($Nu_{h}$) in Eq. (30) is associated with $\theta '(1)$, therefore, the rate of heat transfer at wall is represented by $\theta '(1)$ and it can be graphed against different parameters, which are involved in the final system of BVP of ODEs and BCs. Similarly, the mass flux or rate of mass transfer is defined by Fick’s Law as:

31 $$\begin{aligned} \mathbf { q_{m}}= -D_{b}\nabla C= -D_{b}\Big(\frac{\partial C}{\partial x}, \frac{\partial C}{\partial y}\Big) = -\frac{D_{b} C_{0}}{h^4}[-m(3 \phi (\eta )+\eta \phi '(\eta )), \phi '(\eta )]. \end{aligned}$$

The dimensionless form of $\mathbf {q_m}$ gives the Sherwood Number $(Sh_{h})$ as:

32 $$\begin{aligned} Sh_{h}=\frac{D_{b}}{h}[-m(3+ \phi '(1)), \phi '(1)]. \end{aligned}$$

Note that in either case, the Sherwood number ($Sh_h$) in Eq. (32) is associated with $\phi '(1)$, therefore, the rate of mass transfer at wall is represented by $\phi '(1)$ and it can be graphed against different parameters, which are involved in the final system of BVP of ODEs and BCs.

**Figure 12:**
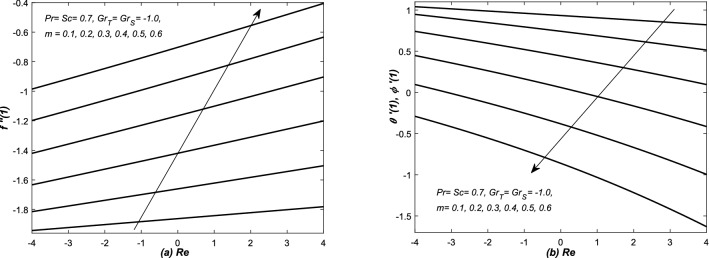
(**a**) Skin friction coefficient $f''(1)$ and (**b**) heat and mass transfer coefficients are plotted against Reynolds number *Re* for opposing flow, whereas, different values are assigned to the slope *m*.

**Figure 13:**
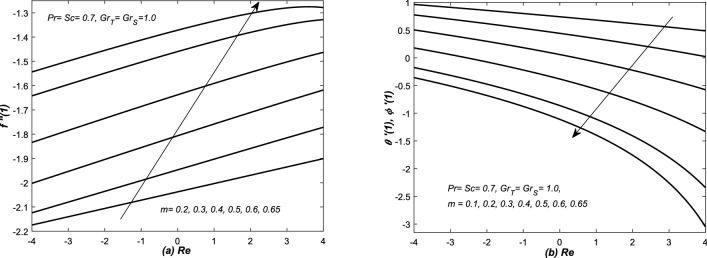
(**a**) Skin friction coefficient $f''(1)$ and (**b**) heat and mass transfer coefficients are plotted against Reynolds number *Re* for assisting flow, whereas, different values are assigned to the slope *m*.

**Figure 14:**
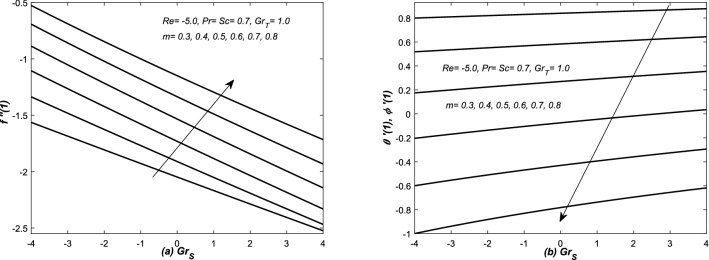
Converging channel: (**a**) Skin friction coefficient $f''(1)$ and (**b**) heat and mass transfer coefficients are plotted against solutal Grashof number $Gr_{S}$ and different values are assigned to the slope *m*.

**Figure 15:**
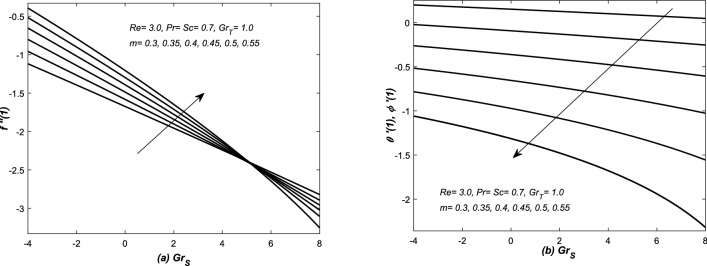
Diverging channel: (**a**) Skin friction $f''(1)$ and (**b**) heat and mass transfer coefficient are plotted against solutal Grashof number $Gr_{S}$ and different values are assigned to the slope *m*.

In Figs. 12, 13, 14 and 15, the skin friction coefficient $f''(1)$, rate of two diffusion functions (i.e. $\theta '(1), \phi '(\eta ))$ at the top surface of the channel are evaluated and graphed against different parameters (i.e. *Re* and $Gr_{S}$). Note that skin friction $(f''(1)),$ two diffusion functions ($\theta '(1),$ and $\phi '(1)$) are found in Eqs. (28, 29, and 31), however, they are evaluated from the solution of system of Eqs. (11–16). In Figs. 12(a,b) and 13(a,b), $f''(1)$ and $\theta '(1)$ along with mass transfer coefficient $\phi '(1)$ are plotted against *Re* for various values of *m*. Both assisting and opposing flows have been taken under consideration in these figures. The linear profiles of $f''(1)$ are increasing function of Re and they are increased with increasing of *m*. However, the profiles of $\theta '(1)$ and $\phi (1)$ are linear (nonlinear) for small (large) values of *m*. Whereas, they are decreasing function of *Re* and decreased with the increasing value of *m*. The non-linearity in the profiles of $\theta '(1)$ and $\phi '(1)$ has been observed for a little bit large value of *m*.

In Figs. 12b, 13, 14 and 15b, three types variations occurred in the profiles of for the diffusion variables. The diffusion rates are uniform for small value of *m* in case of both converging and diverging flows. In case of converging flow, it is linear and increasing function of $Gr_{S}$ and decreased with the increasing value of *m*. For diverging flow, it is decreasing function of $Gr_{S}$ and decreased with the increasing value of *m*. Moreover, in this case, the profiles of diffusion rates are non-linear for large values of *m*.

In Figs. 14a and 15a, $f''(1)$ is graphed against $Grx_{S}$ in the presence of both converging and diverging flows. In both these figures, the linear profiles of $f''(1)$ are decreasing function of $Gr_{S}$ for both converging and diverging flows and increased with the increasing values of *m*. In Fig. 14a, the profiles of $f''(1)$ suddenly decreased against $Gr_{S}$ for small *m*. In Fig. 15a, the profiles of $f''(1)$ intersect each other at $f''(1)= -2.5$.

## Conclusion

Double-diffusive convection in flow is examined inside a converging (diverging) channel of rectangular, inclined and heated walls. Whereas, the concluding remarks of the whole analysis have been presented here:

1. The present investigations have been compared with the classical analysis of flow in converging and diverging channels. Whereas, the published results have been retrieved easily from the current modeled problem. The curves of skin friction against modified Reynold’s number $Re^{*}$ is relatively close to a straight line for negative values of $Re^{*}$. For $Re^{*}>0$, the profile of $-f''(1)$ has reached to zero value at $Re^{*}\simeq 10.31$. The dimensionless velocity profiles plotted against the similarity variable for modified Reynold’s number $Re^{*}$, are compared with the published work of Millsaps and Pohlhausen given in White^24^, Laila et al.^25^ and Marwat et al.^26^ and exactly the same results have obtained.
2. The thermal and solutal Grashof numbers $(Gr_{T}, Gr_{S})$ have assisted and opposed the flows inside a channel depending upon the values of these two Grashof numbers, nature of the fluid and type of the channel, and its walls inclination. Moreover, the significant changes in velocity profile create reasonable variation in the temperature and concentration functions. Velocity is increased (decreased) for assisting (opposing) flows in such situations.
3. In case of converging (diverging) flow, both the diffusion distributions have been decreased (increased) uniformly with the increase of $Gr_{T}$ and $Gr_{S}$. The two diffusion boundary layers have also appeared in the surrounding of the centre and upper wall of the channel. The sudden rise in the diffusion variables has been noted at the centre of the channel for converging flow.
4. The thermal and solutal Grashof numbers have minor effects on the streamlines near the centre of the channel inside both converging and diverging flows. The profiles of stream lines slightly vary in converging flow. However, these variations are prominent in diverging flow. Furthermore, the behavior of streamlines is identical at the center of channel for the given set of parameters values.
5. The skin friction coefficient has decreased against $Gr_{S}$ for both converging and diverging flows, however, linear profiles of $f''(1)$ have been observed in all cases, whereas, it is an increasing function of *m*.
6. In case of converging flow, the profiles of rates of heat and mass transfer i.e. $\theta '(1)$ and $\phi '(1)$ against $Gr_{S}$ are linear for small *m*. Moreover, for diverging flow and small *m*, they are linear against $Gr_{S}$. The rates of heat and mass transfer are uniform for both converging and diverging flows and small *m*.

## List of symbols

*x*, *y*: Cartesian Coordinates
*t*: time variable
*u*, *v*: velocity components
*p*: pressure distribution variable
$c_p$: specific heat at constant pressure
$D_b$: mass diffusivity coefficient
*U*(*x*): velocity at the centre of channel
$U_0$: uniform free stream velocity
*h*(*x*): half spacing between walls
$h_{0}$: half spacing between walls
*m*: slope of the channel’s upper walls
*T*: temperature variable
$T_{w}(x)$: temperature at wall
$T_0$: temperature at the channel’s centre
$T_1$: controlling parameter for wall’s temperature
*C*: species concentration variable
$C_w(x)$: species concentration at the wall
$C_{0}$: concentration at the channel’s centre
$C_1$: controlling parameter for wall’s concentration
$C_f$: skin friction coefficient
$g_{x}$, $g_{y}$: gravitational acceleration’s components
$\mathbf {q_w}$: surface heat flux
$$\mathbf {q_m}$$: mass flux
$a_{n}, b_{n},c_{n}, d_{n}; n \epsilon {\mathbf {W}}$: perturbation solution’s constant

## Dimensionless functions

$f(\eta )$: dimensionless stream function
$$\theta (\eta )$$: dimensionless temperature
$\phi (\eta )$: dimensionless concentration function
$$P(\eta )$$: dimensionless pressure
*P*(1): dimensionless pressure at the top wall

## Subscripts

*a*: analytical value
*n*: numerical value
*w*: wall
*S*: solutal
*T*: thermal

## Dimensionless numbers

*Re*: Reynolds number
$Re^*=mRe$: modified Reynolds number
*Pr*: Prandtl number
*Sh*: Sherwood number
*Nu*: Nusselt number
$Nu_{h}$: Nusselt number defined at top wall
$Grx_{T}$: Thermal Grashof number along x-axis
$Gry_{T}$: Solutal Grashof number along y-axis
$Grx_{S}$: Solutal Grashof number along x-axis
$Gry_{S}$: Solutal Grashof number along y-axis

## Greek letters

$$\alpha $$: thermal diffusivity
$\beta$: thermal expansion coefficient
$\beta ^{*}$: volumetric coefficient of mass transfer
$$\gamma = \tan ^{-1}(m)$$: inclination angle
$$\eta $$: similarity variable
$\rho$: density of the fluid
$$\tau $$: shear stress
$\chi =\frac{x}{h_{0}}$: dimensionless axial space variable
$$\kappa $$: thermal conductivity
$$\mu $$: dynamic viscosity
$$\nu $$: kinematic viscosity
$$\omega $$: vorticity function
$\zeta$: dimensionless heat transfer
$$\Delta T$$: temperature difference
$$\Delta C$$: concentration difference
$$\Delta f$$: $$ f_{n}- f_{n}$$
$$\Delta \theta $$: $$ \theta _{n} - \theta _{a} $$
$$\Delta \phi $$: $$ \phi _{n}- \phi _{n}$$

## References

[1] Pop II, Ingham DB (2001). Convective Heat Transfer: Mathematical and Computational Modeling of Viscous Fluids and Porous Media.

[2] Mojtabi,A., & Charrier-Mojtabi, M. C., Double diffusive convection in porous media. In *Handbook of Porous Media*, 2nd edn. 270-320 (2005).

[3] Stern ME (1960). The salt fountain and thermohaline convection. Tellus.

[4] Stern ME (1969). Collective instability of salt fingers. J. Fluid Mech..

[5] Somers EV (1956). Theoretical considerations of combined thermal and mass transfer from a vertical flat plate. J. Appl. Mech..

[6] Gebhart B, Pera L (1971). The nature of vertical natural convection flows resulting from the combined buoyancy effects of thermal and mass diffusion. Int. J. Heat Mass Transf..

[7] Nield DA (1968). Onset of thermohaline convection in a porous medium. Water Resour. Res..

[8] Baines PG, Gill AE (1969). On thermohaline convection with linear gradients. J. Fluid Mech..

[9] Guo J, Qin Y, Kaloni PN (1994). Non-linear stability problem of a rotating doubly diffusive fluid layer. Int. J. Eng. Sci..

[10] Khanafer K, Vafai K (2002). Double-diffusive mixed convection in a lid-driven enclosure filled with a fluid-saturated porous medium. Numer. Heat Transf. Part A.

[11] Sunil Sharma A, Sharma RC (2006). Effect of dust particles on ferrofluid heated and soluted from below. Int. J. Therm. Sci..

[12] Gaikwad SN, Malashetty MS, Prasad KR (2009). An analytical study of linear and nonlinear double-diffusive convection in a fluid saturated anisotropic porous layer with Soret effect. Appl. Math. Model..

[13] Cheng P (1977). Similarity solutions for mixed convection from horizontal impermeable surfaces in saturated porous media. Int. J. Heat Mass Transf..

[14] Cheng P (1985). Natural Convection in a Porous Medium: External Flow. Natural Convection: Fundamentals and Applications.

[15] Nield DA, Bejan A (2010). Convection in Porous Media.

[16] Ingham DB, Pop I (2002). Transport Phenomenon in porous media.

[17] Yih KA (1999). Coupled heat and mass transfer by free convection over a truncated cone in porous media. Acta Mech..

[18] Chamkha AJ (2001). Coupled heat and mass transfer by natural convection about a truncated cone in the presence of magnetic field and radiation effects. Numer. Heat Transf. Part-A.

[19] Narayana, & Sibanda, P. On the solution of double-diffusive convective flow due to a cone by a linearization method. *J. Appl. Math.* **2012**, 1–19 (2012).

[20] Hyun JM, Lee JW (1990). Double-diffusive convection in a rectangle with cooperating horizontal gradients of temperature and concentration. Int. J. Heat Mass Transf..

[21] Lee JW, Hyun JM (1990). Double-diffusive convection in a rectangle with opposing horizontal temperature and concentration gradients. Int. J. Heat Mass Transf..

[22] Bilgen E, Vasseur P, Mamou M (1995). Multiple solutions for double-diffusive convection in a vertical porous enclosure. Int. J. Heat Mass Transf..

[23] Mortimer RG, Eyring H (1980). Elementary transition state theory of the Soret and Dufour effects. Proc. Natl. Acad. Sci. U.S.A..

[24] White FM (2006). Viscous Fluid Flow.

[25] Laila R, Marwat DNK, Ali A (2021). Flow and heat transfer in a rectangular converging (diverging) channel: New formulation. J. Egypt. Math. Soc..

[26] Nawaz KMD, Asghar S, Ali A (2017). Flow between two rectangular inclined plane walls. Chin. J. Phys..

